# Caries

**Published:** 1893-01

**Authors:** J. W. Wassall


ARTICLE III.

### CARIES.

Generally speaking, the whole range of pathological conditions to which the teeth are subject, may be classified under two heads:

1. Diseases affecting the crowns of the teeth.
2. Diseases affecting the roots and socket.

Except traumatic lesions, both these classes of disease are preventable by conscientiously following strict rules of hygiene. An attempt will be made later on to formulate these rules.

It requires no argument to persuade you of the truth of the hypothesis that "the disease known as dental caries will not occur except where the causal microbes are permitted to grow undisturbed on the teeth." The rational conclusion is, then, that if the teeth are not allowed to accumulate deposits on either their exposed or protected surfaces, they will be exempt from caries. More broadly stated the proposition is :

Given the varying predispositions of different individuals to caries, which is governed by the laws of heredity and environment, the growth of micro-organisms in the mouth is in proportion to the amount of disturbance they suffer, or rest and opportunity they enjoy. That is a recognized fact, and almost axiomatic.

Is the practice of dentists as followed in the daily routine of seeing patients consistent with these undisputed etiological facts ? We all know it is not.

Plainly, then, one great step in the direction of the establishing of correct habits of cleansing the teeth in the general public is to be accomplished by reform of the dentist, making obligatory on him the performance of his duty. Similarly, as public sentiment now requires a man to be a graduate of a reputable college before entering on practice, so can it compel him to teach oral hygiene and require his patients to observe its rules.

For the purpose of convenience, allow me to place people in three classes with respect to the care they give their teeth. A few people by assiduous, intelligent care and a natural tendency to cleanliness to the teeth themselves, have clean teeth from year to year; the proportion is very small indeed, say 1 per cent. A second class are just as anxious and spend as much time, or perhaps more, but their efforts are ineffective because misdirected and on account of unsuitable cleansing implements and materials. But most people give to their teeth slovenly care.

If we are justified in assuming that caries and pyorrhea alveolaris, the two diseases most destructive to the teeth, only occur in the presence of foreign matter which is allowed to accumulate on them, we next want to know how to prevent the accumulations.

My own belief is, that it is possible in most cases for the individual to keep the teeth free from all deposits and food *debris*. I have been led to this belief by the study of several cases which have been under my care and observation for some time past.

At the first appointment examine the mouth as to the degree of cleanliness usual to the person, and make a note of the conditions in the record book for future reference. Catechise the person to ascertain what are the present habits as to the number of brushings per diem, dentifrices, floss, tooth-pick, etc. Make notes of each point. Then show to the person with mirror every part of the teeth which is unclean. The patient may apologetically, or even indignantly, say that it is impossible to be more thorough than he is. It is not prudent to deny this at the time if you wish to accomplish your purpose, unless to explain that you may be able to help him to be more successful. You must gain his good will and inspire the desire by giving arguments for ordinary (ordinary in its new sense) cleanliness, and explaining its benefits.

It is now proper to scale and polish perfectly each tooth. The mirror should now be used again to show the patient the change. Now is your time to teach a lesson of the most forcible kind. Say to him, "It is quite possible for you, by your own efforts, to keep every tooth in your mouth absolutely clean." Make this statement positively and dogmatically. With the effrontery, if you please, of a bichloride of gold doser promising his dupe a sure cure. The moral effect of such an assurance and the placing of the responsibility where it belongs will be very helpful.

The instructions in the daily care to be given the teeth should now be distinctly and carefully impressed on the patient's mind. Printed directions for children might be useful, but I have never tried them.

Now, you may say all this and more—some people will acquire the habit easily—others, never. It is your business to labor patiently to the right end.

At the next appointment, for a filling, perhaps, make inquiries to see that your instructions have been followed.

If they have not, in every detail, reassert the necessity for it, refreshing the patient's mind, and, in some cases, demonstrate with a brush the practicability of your statments. Be careful not to lay down more rules than can be followed.

This system of following up the matter as long as the appointment lasts, seeing to it that the patient's efforts are sustained and effective, and that the habit is formed, are the most important and valuable services you can render in your capacity as a dentist.

The task is not a thankless one; your labor will invariably be highly appreciated, and as these patients come back to you from time to time, it will be one of the greatest satisfaction of your professional experience to be able to say, after the most conscientious examination with the most searching of fine explorers, that you find no decay—no pockets.

Now as to rules: Of first importance, of course, is the use of the brush. Twice daily is sufficient—at night before retiring and in the morning. It is of the utmost advantage to have three brushes in use. This is imperatively required—*three brushes*. A brush will not do effective work unless it has time to dry out. The bristles will always be too soft if it is used more than once in twenty-four hours. More good is obtained by this than one would expect without trying. What is known to the dealers as "medium" grade of stiffness is the best. To the well kept mouth with all its parts and members in a healthy state, a good vigorous brushing with a moderately stiff brush is a pleasurable sensation. Three brushes in use at the same time will also give longer service than if bought consecutively.

The usual instructions as to vertical movements should be given. Special directions are necessary to make the patient reach all the accessible surface. Remember that few people do this. It becomes your duty to dispel the common delusion that the last molar and lingual surfaces cannot be brushed. Any one can touch all these surfaces who will use his brain, and for a time watch himself during the process. The old slovenly or thoughtless habit is to be broken up and a new set of movements learned. Insist on thoroughness, and keep on insisting.

Dentifrices: Some form of powder is the only proper dentifrice, and it should always be used whenever the brush is used.

A pound can or bottle from which the small bottle for present use may be replenished is indispensable in preventing a giving out of the supply and a consequent deposit of calculus by a few days' forgetfulness to go to the druggist. Aim to make the powder as agreeable as possible, especially for children.

Women and children can be induced to use floss silk or rubber bands for interdental spaces at least once a day. Men should be encouraged to use the tooth-pick.

Disinfectant and antiseptic washes are allowable and useful, but should not take the place of powder applied by brush at the stated intervals. As a rule I recommend it only in special cases. The best results are obtained by the simplest means faithfully employed. It is more than probable that the individual whom we have in hand will have anywhere from three to a dozen appointments for the other needed operations. Opportunities are thus afforded to assist in putting him on the right track. You have given him a good start—how shall he be watched?

One or two months later, by previous arrangement, he must be summoned to your office for inspection. Perhaps—yes, usually—a little scolding and lecturing will be necessary to put him right. At suitable intervals he must be notified again, and both of you will be delighted at the fine appearance and freedom from disease presented.

My own experience is that it is not a difficult matter to bring about this happy condition, and one success pays for a score of failures.

RESUME.

A statement to the patient of the advantages and desirability of absolute cleanliness of the teeth.

Namely, that it affords immunity from caries of crown and loosening of tooth in socket.

Economy of time.

Economy of money.

Economy of pain, and preservation of natural teeth all through life.

A statement of the means to be employed to maintain absolute cleanliness of the teeth.

Establishment of habit.

Brushing the teeth three times a day.

Thorough brushing of labial and lingual surfaces twice daily with powder.

Silk, rubber bands or pick.

Regular visitation by appointments for inspection and instruction.—J. W. WASSALL, in *Dental Review.*

